# Crystal structure of 5-(5-chloro-2-hydroxy­benzo­yl)-2-(2-methyl-1*H*-indol-3-yl)nicotino­nitrile

**DOI:** 10.1107/S2056989015018058

**Published:** 2015-10-07

**Authors:** G. Vimala, N. Poomathi, Y. AaminaNaaz, P. T. Perumal, A. SubbiahPandi

**Affiliations:** a Department of Physics, Presidency College (Autonomous), Chennai 600 005, India; b Organic Chemistry, CSIR–Central Leather Research Institute, Adyar, Chennai 600 020, India

**Keywords:** crystal structure, nicotino­nitrile, acrylate derivatives, indole unit, N—H⋯N hydrogen bonds, C—H⋯π inter­actions, π–π inter­actions

## Abstract

In the title compound, C_22_H_14_ClN_3_O_2_, the indole unit is essentially coplanar, with a maximum deviation of 0.035 Å for the C atom bearing the methyl group. The central pyridine ring is inclined to the indole ring system by 43.7 (1)°. The dihedral angle between the phenyl ring and the indole ring system is 15.7 (2)°, while that between the phenyl ring and the central pyridine ring is 46.3 (1)°. The mol­ecular structure is stabilized by an intra­molecular O—H⋯O hydrogen bonding, forming an *S*(6) ring motif. In the crystal, mol­ecules are linked *via* pairs of N—H⋯N hydrogen bonds, forming inversion dimers with an *R*_2_^2^(16) ring motif. The crystal structure also features C—H⋯π and π–π inter­actions [centroid–centroid separation = 3.688 (1) Å].

## Related literature   

For applications of acrylate derivatives, see: Barden (2011 ▸); Chai *et al.* (2006 ▸); Nieto *et al.* (2005 ▸); Singh *et al.* (2000 ▸); Andreani *et al.* (2001 ▸); Quetin-Leclercq (1994 ▸); Mukhopadhyay *et al.* (1981 ▸). For related crystal structures, see: Penthala *et al.* (2008 ▸). For graph-set analysis, see: Grell *et al.* (2000 ▸). 
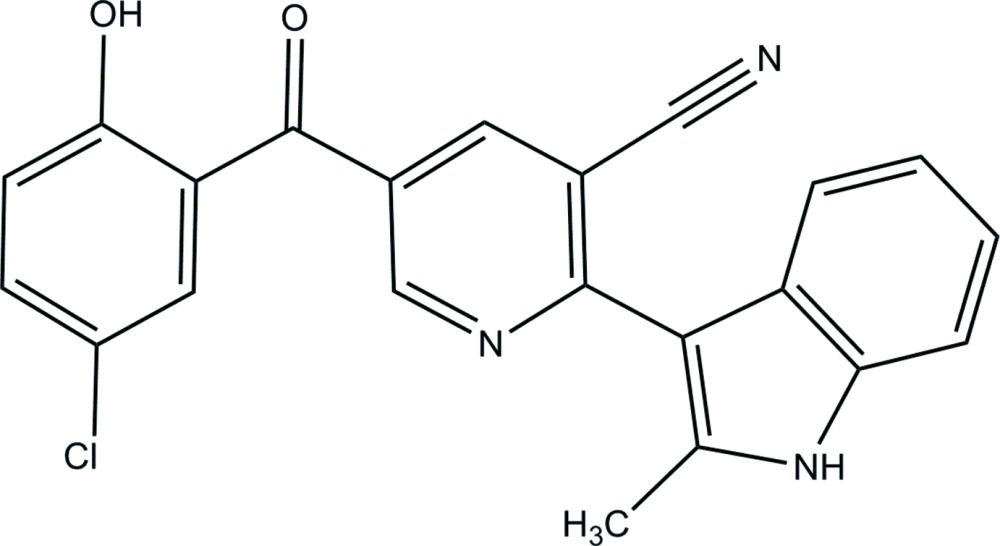


## Experimental   

### Crystal data  

C_22_H_14_ClN_3_O_2_
*M* *_r_* = 387.81
Monoclinic 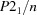

*a* = 16.0673 (15) Å
*b* = 7.4804 (7) Å
*c* = 17.0159 (15) Å
β = 113.452 (3)°
*V* = 1876.2 (3) Å^3^
*Z* = 4
Mo *K*α radiation
μ = 0.23 mm^−1^
*T* = 293 K
0.27 × 0.23 × 0.18 mm

### Data collection  

Bruker Kappa APEXII CCD diffractometer
Absorption correction: multi-scan (*SADABS*; Bruker, 2008 ▸) *T*_min_ = 0.941, *T*_max_ = 0.960
7690 measured reflections
3638 independent reflections
2447 reflections with *I* > 2σ(*I*)
*R*_int_ = 0.038

### Refinement  

*R*[*F* ^2^ > 2σ(*F* ^2^)] = 0.057
*wR*(*F* ^2^) = 0.170
*S* = 1.01
3638 reflections
254 parameters
H-atom parameters constrained
Δρ_max_ = 0.43 e Å^−3^
Δρ_min_ = −0.36 e Å^−3^

### 

Data collection: *APEX2* (Bruker, 2008 ▸); cell refinement: *APEX2* and *SAINT* (Bruker, 2008 ▸); data reduction: *SAINT* and *XPREP* (Bruker, 2008 ▸); program(s) used to solve structure: *SHELXS97* (Sheldrick, 2008 ▸); program(s) used to refine structure: *SHELXL97* (Sheldrick, 2008 ▸); molecular graphics: *ORTEP-3 for Windows* (Farrugia, 2012 ▸); software used to prepare material for publication: *PLATON* (Spek, 2009 ▸).

## Supplementary Material

Crystal structure: contains datablock(s) global, I. DOI: 10.1107/S2056989015018058/zp2018sup1.cif

Structure factors: contains datablock(s) I. DOI: 10.1107/S2056989015018058/zp2018Isup2.hkl

Supporting information file. DOI: 10.1107/S2056989015018058/zp2018Isup3.cml

**Fig. 1.**
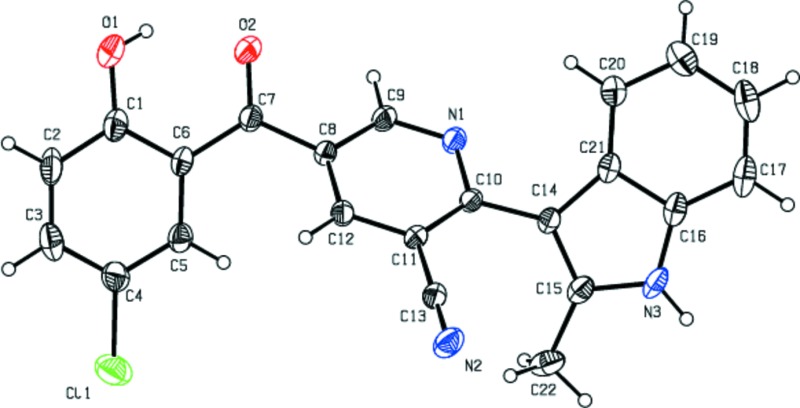
The molecular structure of the title compound, with the atomic numbering scheme and displacement ellipsoids drawn at 30% probability level.

**Fig. 2.**
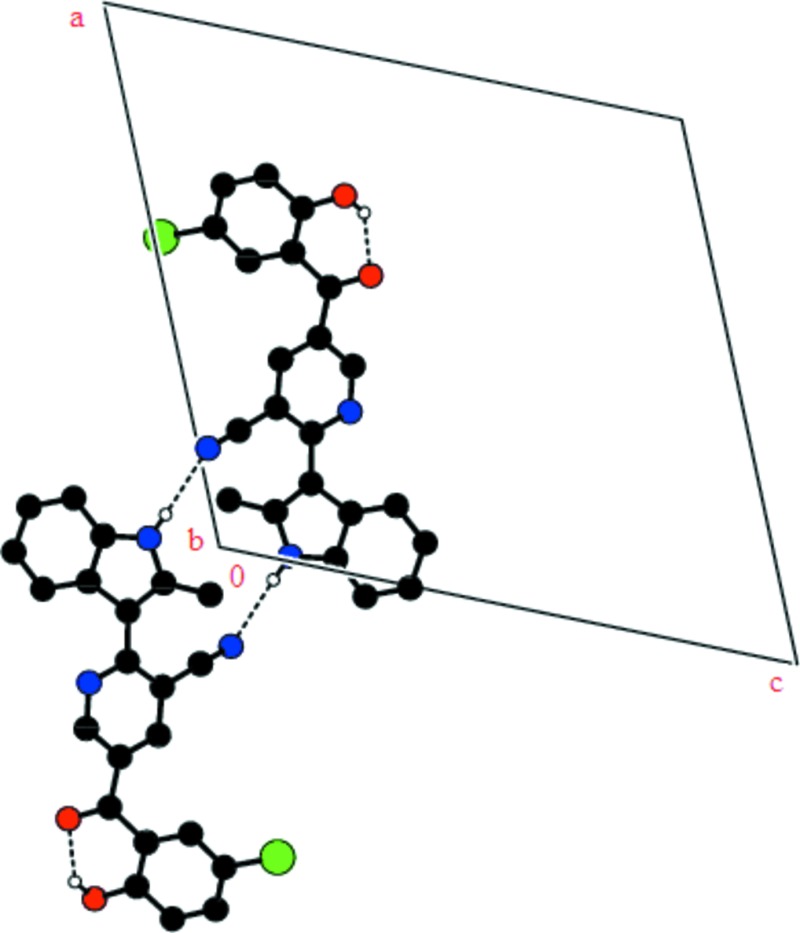
O—H···O intra and N—H···N intermlecular interactions (dotted lines) in the crystal structure of the title compound. The crystal packing of the molecules is viewed down the b axis.

. DOI: 10.1107/S2056989015018058/zp2018fig1.tif
The mol­ecular structure of the title compound, with the atomic numbering scheme and displacement ellipsoids drawn at 30% probability level.

b . DOI: 10.1107/S2056989015018058/zp2018fig2.tif
O—H⋯O intra and N—H⋯N inter­mlecular inter­actions (dotted lines) in the crystal structure of the title compound. The crystal packing of the mol­ecules is viewed down the *b* axis.

CCDC reference: 1427861

Additional supporting information:  crystallographic information; 3D view; checkCIF report

## Figures and Tables

**Table 1:** Hydrogen-bond geometry (, ) *Cg*3 and *Cg*4 are the centroids of the C1C6 and C16C21 rings, respectively.

*D*H*A*	*D*H	H*A*	*D* *A*	*D*H*A*
O1H1O2	0.82	1.91	2.596(3)	140
N3H3*A*N2^i^	0.86	2.27	3.110(4)	164
C2H2*Cg*3^ii^	0.90	2.93	3.656(4)	136
C12H12*Cg*4^iii^	0.93	2.99	3.361(4)	106

Symmetry codes: (i) 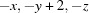; (ii) 
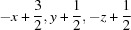
; (iii) 
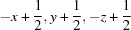
.

## supplementary crystallographic information

### S1. Comment

In modern times, analogs based on indole are significant players in a diverse array of markets such as dyes, plastics, agriculture, vitamin supplements, over-the-counterdrugs, flavour enhancers and perfumery (Barden, 2011). Indole derivatives exhibit antihepatitis B virus (Chai *et al.*, 2006) and antibacterial (Nieto *et al.*, 2005) activities. Indole derivatives have been found to exhibit antibacterial, antifungal (Singh *et al.*, 2000) and antitumour activities (Andreani *et al.*, 2001). Some of the indole alkaloids extracted from plants possess interesting cytotoxic, antitumour or antiparasitic properties (Quetin-Leclercq, 1994; Mukhopadhyay *et al.*, 1981). Against this background, the crystal structure of the title compound has been determined and the results are presented herein.

In the title molecule, the indole unit is essentially co-planar with a maximum deviation of -0.035 Å for the C15 atom. The central pyridine (N1/C8—C12) ring is almost halfway to be orthogonal to the indole ring system (N3/C14—C21), making a dihedral angle of 43.7 (1)°. The carbonyl-bound phenyl ring (C16—C21) forms a dihedral angle of 15.7 (2)° with the plane of the indole ring system. The pyridine ring and the phenyl ring are inclined at an angle of 46.3 (1)°. The cyano bond distance C13≡N2 agrees well with the reported value of 1.141 (4) Å.

The crystal packing (Fig. 2 and Table 1) is stabilized by an intramolecular O—H···O hydrogen bond, forms S(6) ring motif. The molecules are linked into inversion dimers *via* N—H···N hydrogen bonds resulting in an *R*^2^_2_(16) graph-set motif, which are stablized by C—H···π (Table 1) and π-π interactions. The *Cg*1···*Cg*2^ii^ seperation is 3.688 (1) Å (Fig.2; *Cg*1 and *Cg*2 are centroids of the N3/C14—C16/C21 ring and N1/C8—C12 pyridine ring, repectively; symmetry codes: (ii) 1/2 - *x*, *y* - 1/2, 1/2 - *z*).

### S2. Experimental

A mixture of 6-chlorol-3-formylchromone (1 mmol), cyanoacetylindole (1 mmol) and ammonium acetate (1 mmol) in DMF and a catalytic amount of SnCl2.2H2O (0.020 mol %) was added and refluxed for about 3 hrs. After completition of the reaction, the solvent was removed under reduced pressure and the residue was purified by column chromatography on siliga gel (3:97% ethylacetate and petether) to afford pure product. The purified compound was recrystalized from ethanol by using slow evaporation method. The yield of the isolated product was 92%, giving block like crystals suitable for *X* ray diffraction.

### S3. Refinement

All H atoms were fixed geometrically and allowed to ride on their parent C atoms, with C—H distances fixed in the range 0.93–0.97 Å with *U*_iso_(H) = 1.5*U*_eq_(C) for methyl H atoms and 1.2*U*_eq_(C) for all other H atoms.

### Crystal data

**Table:**

C_22_H_14_ClN_3_O_2_	*F*(000) = 800
*M**_r_* = 387.81	*D*_x_ = 1.373 Mg m^−^^3^
Monoclinic, *P*2_1_/*n*	Mo *K*α radiation, λ = 0.71073 Å
Hall symbol: -P 2yn	Cell parameters from 2447 reflections
*a* = 16.0673 (15) Å	θ = 1.5–25.9°
*b* = 7.4804 (7) Å	µ = 0.23 mm^−^^1^
*c* = 17.0159 (15) Å	*T* = 293 K
β = 113.452 (3)°	Block, colourless
*V* = 1876.2 (3) Å^3^	0.27 × 0.23 × 0.18 mm
*Z* = 4	

### Data collection

**Table:**

Bruker Kappa APEXII CCD diffractometer	3638 independent reflections
Radiation source: fine-focus sealed tube	2447 reflections with *I* > 2σ(*I*)
Graphite monochromator	*R*_int_ = 0.038
Detector resolution: 0 pixels mm^-1^	θ_max_ = 25.9°, θ_min_ = 1.5°
ω and φ scan	*h* = −19→15
Absorption correction: multi-scan (*SADABS*; Bruker, 2008)	*k* = −7→9
*T*_min_ = 0.941, *T*_max_ = 0.960	*l* = −20→20
7690 measured reflections	

### Refinement

**Table:**

Refinement on *F*^2^	Primary atom site location: structure-invariant direct methods
Least-squares matrix: full	Secondary atom site location: difference Fourier map
*R*[*F*^2^ > 2σ(*F*^2^)] = 0.057	Hydrogen site location: inferred from neighbouring sites
*wR*(*F*^2^) = 0.170	H-atom parameters constrained
*S* = 1.01	*w* = 1/[σ^2^(*F*_o_^2^) + (0.0793*P*)^2^ + 1.1008*P*] where *P* = (*F*_o_^2^ + 2*F*_c_^2^)/3
3638 reflections	(Δ/σ)_max_ < 0.001
254 parameters	Δρ_max_ = 0.43 e Å^−^^3^
0 restraints	Δρ_min_ = −0.36 e Å^−^^3^

### Special details

**Table:**

Geometry. All e.s.d.'s (except the e.s.d. in the dihedral angle between two l.s. planes) are estimated using the full covariance matrix. The cell e.s.d.'s are taken into account individually in the estimation of e.s.d.'s in distances, angles and torsion angles; correlations between e.s.d.'s in cell parameters are only used when they are defined by crystal symmetry. An approximate (isotropic) treatment of cell e.s.d.'s is used for estimating e.s.d.'s involving l.s. planes.
Refinement. Refinement of *F*^2^ against ALL reflections. The weighted *R*-factor *wR* and goodness of fit *S* are based on *F*^2^, conventional *R*-factors *R* are based on *F*, with *F* set to zero for negative *F*^2^. The threshold expression of *F*^2^ > σ(*F*^2^) is used only for calculating *R*-factors(gt) *etc*. and is not relevant to the choice of reflections for refinement. *R*-factors based on *F*^2^ are statistically about twice as large as those based on *F*, and *R*- factors based on ALL data will be even larger.

### Fractional atomic coordinates and isotropic or equivalent isotropic displacement parameters (Å^2^)

**Table:**

	*x*	*y*	*z*	*U*_iso_*/*U*_eq_	
C1	0.6829 (2)	1.0448 (4)	0.2799 (2)	0.0475 (8)	
C2	0.7315 (2)	1.0614 (5)	0.2278 (2)	0.0599 (9)	
H2	0.7877	1.1176	0.2487	0.072*	
C3	0.6961 (2)	0.9945 (5)	0.1459 (2)	0.0608 (9)	
H3	0.7285	1.0055	0.1115	0.073*	
C4	0.6122 (2)	0.9108 (5)	0.11432 (19)	0.0497 (8)	
C5	0.56120 (19)	0.9038 (4)	0.16240 (17)	0.0416 (7)	
H5	0.5036	0.8532	0.1393	0.050*	
C6	0.59480 (17)	0.9718 (4)	0.24590 (17)	0.0377 (6)	
C7	0.54170 (18)	0.9672 (4)	0.29877 (17)	0.0363 (6)	
C8	0.44084 (17)	0.9513 (4)	0.26081 (16)	0.0327 (6)	
C9	0.39945 (18)	0.8749 (4)	0.31059 (17)	0.0382 (6)	
H9	0.4370	0.8306	0.3641	0.046*	
C10	0.25482 (17)	0.9246 (3)	0.21050 (15)	0.0309 (6)	
C11	0.29038 (17)	1.0110 (4)	0.15727 (15)	0.0328 (6)	
C12	0.38420 (17)	1.0235 (4)	0.18271 (16)	0.0341 (6)	
H12	0.4084	1.0794	0.1477	0.041*	
C13	0.23147 (18)	1.0979 (4)	0.07944 (17)	0.0391 (7)	
C14	0.15806 (17)	0.9068 (4)	0.19015 (16)	0.0331 (6)	
C15	0.09024 (19)	0.8498 (4)	0.11465 (17)	0.0411 (7)	
C16	0.02316 (18)	0.9066 (4)	0.20583 (19)	0.0424 (7)	
C17	−0.0377 (2)	0.9216 (5)	0.2447 (3)	0.0577 (9)	
H17	−0.0992	0.8991	0.2146	0.069*	
C18	−0.0039 (2)	0.9706 (5)	0.3289 (3)	0.0625 (10)	
H18	−0.0430	0.9798	0.3569	0.075*	
C19	0.0883 (2)	1.0074 (4)	0.3739 (2)	0.0565 (9)	
H19	0.1091	1.0423	0.4310	0.068*	
C20	0.1487 (2)	0.9931 (4)	0.33560 (18)	0.0428 (7)	
H20	0.2100	1.0177	0.3662	0.051*	
C21	0.11658 (17)	0.9409 (3)	0.25004 (17)	0.0356 (6)	
C22	0.0944 (2)	0.7734 (5)	0.03518 (18)	0.0563 (9)	
H22A	0.0340	0.7473	−0.0054	0.084*	
H22B	0.1296	0.6654	0.0490	0.084*	
H22C	0.1221	0.8583	0.0108	0.084*	
N1	0.31060 (14)	0.8602 (3)	0.28774 (13)	0.0373 (6)	
N2	0.18555 (17)	1.1658 (4)	0.01731 (16)	0.0565 (7)	
N3	0.00999 (15)	0.8531 (3)	0.12427 (16)	0.0481 (7)	
H3A	−0.0417	0.8255	0.0849	0.058*	
O1	0.72328 (15)	1.1053 (4)	0.36103 (15)	0.0673 (7)	
H1	0.6973	1.0640	0.3898	0.101*	
O2	0.58003 (13)	0.9826 (3)	0.37762 (12)	0.0537 (6)	
Cl1	0.57239 (7)	0.81118 (15)	0.01424 (5)	0.0737 (4)	

### Atomic displacement parameters (Å^2^)

**Table:**

	*U*^11^	*U*^22^	*U*^33^	*U*^12^	*U*^13^	*U*^23^
C1	0.0320 (16)	0.0497 (19)	0.0610 (19)	0.0020 (13)	0.0186 (14)	−0.0031 (15)
C2	0.0342 (18)	0.072 (2)	0.083 (2)	−0.0061 (16)	0.0330 (17)	−0.0059 (19)
C3	0.047 (2)	0.073 (2)	0.078 (2)	0.0088 (18)	0.0412 (19)	0.0046 (19)
C4	0.0410 (18)	0.061 (2)	0.0480 (17)	0.0175 (15)	0.0186 (14)	0.0019 (15)
C5	0.0311 (15)	0.0455 (17)	0.0471 (16)	0.0076 (13)	0.0143 (13)	0.0030 (13)
C6	0.0244 (14)	0.0397 (15)	0.0473 (16)	0.0027 (12)	0.0125 (12)	0.0018 (12)
C7	0.0276 (14)	0.0419 (16)	0.0384 (15)	0.0000 (12)	0.0121 (12)	0.0023 (12)
C8	0.0249 (13)	0.0380 (15)	0.0340 (13)	0.0006 (11)	0.0104 (11)	−0.0015 (11)
C9	0.0284 (14)	0.0479 (17)	0.0333 (14)	0.0022 (12)	0.0072 (11)	0.0052 (12)
C10	0.0264 (13)	0.0334 (14)	0.0303 (13)	−0.0008 (11)	0.0084 (11)	−0.0012 (10)
C11	0.0272 (14)	0.0383 (14)	0.0295 (13)	0.0006 (11)	0.0078 (11)	0.0003 (11)
C12	0.0287 (14)	0.0394 (15)	0.0357 (14)	−0.0006 (11)	0.0143 (11)	0.0017 (11)
C13	0.0300 (15)	0.0510 (17)	0.0354 (15)	−0.0029 (13)	0.0119 (12)	0.0032 (13)
C14	0.0257 (14)	0.0356 (14)	0.0356 (14)	−0.0015 (11)	0.0098 (11)	0.0032 (11)
C15	0.0326 (16)	0.0429 (16)	0.0404 (15)	−0.0040 (12)	0.0067 (12)	0.0056 (12)
C16	0.0290 (15)	0.0383 (16)	0.0590 (18)	0.0020 (12)	0.0164 (13)	0.0113 (13)
C17	0.0327 (17)	0.054 (2)	0.091 (3)	0.0040 (14)	0.0296 (18)	0.0153 (18)
C18	0.057 (2)	0.057 (2)	0.096 (3)	0.0111 (17)	0.053 (2)	0.0110 (19)
C19	0.067 (2)	0.0496 (19)	0.066 (2)	0.0045 (17)	0.0405 (18)	0.0023 (16)
C20	0.0400 (17)	0.0411 (16)	0.0504 (17)	−0.0008 (13)	0.0213 (14)	0.0007 (13)
C21	0.0255 (13)	0.0325 (14)	0.0478 (16)	0.0006 (11)	0.0134 (12)	0.0066 (12)
C22	0.058 (2)	0.058 (2)	0.0391 (16)	−0.0120 (16)	0.0050 (15)	−0.0068 (14)
N1	0.0286 (12)	0.0456 (14)	0.0353 (12)	−0.0018 (10)	0.0102 (10)	0.0055 (10)
N2	0.0393 (15)	0.074 (2)	0.0440 (15)	−0.0056 (13)	0.0042 (12)	0.0157 (14)
N3	0.0230 (13)	0.0574 (16)	0.0514 (15)	−0.0069 (11)	0.0018 (11)	0.0063 (12)
O1	0.0365 (13)	0.0949 (19)	0.0668 (15)	−0.0177 (12)	0.0167 (11)	−0.0186 (14)
O2	0.0325 (11)	0.0819 (16)	0.0400 (11)	−0.0051 (10)	0.0074 (9)	0.0039 (11)
Cl1	0.0757 (7)	0.0959 (8)	0.0522 (5)	0.0250 (5)	0.0283 (5)	−0.0047 (5)

### Geometric parameters (Å, º)

**Table:**

C1—O1	1.349 (4)	C12—H12	0.9300
C1—C2	1.399 (4)	C13—N2	1.140 (3)
C1—C6	1.409 (4)	C14—C15	1.380 (4)
C2—C3	1.373 (5)	C14—C21	1.445 (4)
C2—H2	0.9300	C15—N3	1.363 (4)
C3—C4	1.386 (5)	C15—C22	1.494 (4)
C3—H3	0.9300	C16—N3	1.377 (4)
C4—C5	1.370 (4)	C16—C17	1.386 (4)
C4—Cl1	1.731 (3)	C16—C21	1.409 (4)
C5—C6	1.399 (4)	C17—C18	1.365 (5)
C5—H5	0.9300	C17—H17	0.9300
C6—C7	1.466 (4)	C18—C19	1.398 (5)
C7—O2	1.239 (3)	C18—H18	0.9300
C7—C8	1.491 (4)	C19—C20	1.372 (4)
C8—C12	1.387 (4)	C19—H19	0.9300
C8—C9	1.391 (4)	C20—C21	1.393 (4)
C9—N1	1.327 (3)	C20—H20	0.9300
C9—H9	0.9300	C22—H22A	0.9600
C10—N1	1.351 (3)	C22—H22B	0.9600
C10—C11	1.405 (4)	C22—H22C	0.9600
C10—C14	1.458 (3)	N3—H3A	0.8600
C11—C12	1.396 (3)	O1—H1	0.8200
C11—C13	1.441 (4)		
			
O1—C1—C2	117.2 (3)	N2—C13—C11	179.1 (3)
O1—C1—C6	123.0 (3)	C15—C14—C21	107.2 (2)
C2—C1—C6	119.8 (3)	C15—C14—C10	128.4 (2)
C3—C2—C1	120.0 (3)	C21—C14—C10	124.3 (2)
C3—C2—H2	120.0	N3—C15—C14	108.5 (3)
C1—C2—H2	120.0	N3—C15—C22	120.0 (3)
C2—C3—C4	120.3 (3)	C14—C15—C22	131.1 (3)
C2—C3—H3	119.9	N3—C16—C17	130.5 (3)
C4—C3—H3	119.9	N3—C16—C21	107.1 (2)
C5—C4—C3	120.4 (3)	C17—C16—C21	122.3 (3)
C5—C4—Cl1	119.8 (3)	C18—C17—C16	117.4 (3)
C3—C4—Cl1	119.8 (2)	C18—C17—H17	121.3
C4—C5—C6	120.8 (3)	C16—C17—H17	121.3
C4—C5—H5	119.6	C17—C18—C19	121.3 (3)
C6—C5—H5	119.6	C17—C18—H18	119.3
C5—C6—C1	118.4 (3)	C19—C18—H18	119.3
C5—C6—C7	122.1 (2)	C20—C19—C18	121.4 (3)
C1—C6—C7	119.5 (3)	C20—C19—H19	119.3
O2—C7—C6	120.2 (2)	C18—C19—H19	119.3
O2—C7—C8	117.5 (2)	C19—C20—C21	118.7 (3)
C6—C7—C8	122.2 (2)	C19—C20—H20	120.6
C12—C8—C9	116.9 (2)	C21—C20—H20	120.6
C12—C8—C7	124.7 (2)	C20—C21—C16	118.8 (3)
C9—C8—C7	118.0 (2)	C20—C21—C14	134.7 (2)
N1—C9—C8	125.1 (2)	C16—C21—C14	106.5 (2)
N1—C9—H9	117.4	C15—C22—H22A	109.5
C8—C9—H9	117.4	C15—C22—H22B	109.5
N1—C10—C11	120.6 (2)	H22A—C22—H22B	109.5
N1—C10—C14	115.5 (2)	C15—C22—H22C	109.5
C11—C10—C14	123.9 (2)	H22A—C22—H22C	109.5
C12—C11—C10	119.8 (2)	H22B—C22—H22C	109.5
C12—C11—C13	119.2 (2)	C9—N1—C10	118.4 (2)
C10—C11—C13	120.9 (2)	C15—N3—C16	110.7 (2)
C8—C12—C11	119.1 (2)	C15—N3—H3A	124.7
C8—C12—H12	120.4	C16—N3—H3A	124.7
C11—C12—H12	120.4	C1—O1—H1	109.5
			
O1—C1—C2—C3	176.8 (3)	C10—C11—C13—N2	−138 (22)
C6—C1—C2—C3	−4.8 (5)	N1—C10—C14—C15	−135.0 (3)
C1—C2—C3—C4	0.1 (5)	C11—C10—C14—C15	48.5 (4)
C2—C3—C4—C5	4.1 (5)	N1—C10—C14—C21	42.3 (3)
C2—C3—C4—Cl1	−174.8 (3)	C11—C10—C14—C21	−134.3 (3)
C3—C4—C5—C6	−3.6 (5)	C21—C14—C15—N3	2.0 (3)
Cl1—C4—C5—C6	175.3 (2)	C10—C14—C15—N3	179.6 (2)
C4—C5—C6—C1	−1.1 (4)	C21—C14—C15—C22	−170.9 (3)
C4—C5—C6—C7	179.7 (3)	C10—C14—C15—C22	6.7 (5)
O1—C1—C6—C5	−176.5 (3)	N3—C16—C17—C18	176.8 (3)
C2—C1—C6—C5	5.3 (4)	C21—C16—C17—C18	−0.3 (4)
O1—C1—C6—C7	2.8 (5)	C16—C17—C18—C19	1.1 (5)
C2—C1—C6—C7	−175.4 (3)	C17—C18—C19—C20	−1.0 (5)
C5—C6—C7—O2	160.6 (3)	C18—C19—C20—C21	0.1 (4)
C1—C6—C7—O2	−18.6 (4)	C19—C20—C21—C16	0.6 (4)
C5—C6—C7—C8	−21.8 (4)	C19—C20—C21—C14	−177.9 (3)
C1—C6—C7—C8	158.9 (3)	N3—C16—C21—C20	−178.3 (2)
O2—C7—C8—C12	144.3 (3)	C17—C16—C21—C20	−0.5 (4)
C6—C7—C8—C12	−33.3 (4)	N3—C16—C21—C14	0.6 (3)
O2—C7—C8—C9	−28.8 (4)	C17—C16—C21—C14	178.4 (3)
C6—C7—C8—C9	153.6 (3)	C15—C14—C21—C20	177.0 (3)
C12—C8—C9—N1	2.6 (4)	C10—C14—C21—C20	−0.7 (5)
C7—C8—C9—N1	176.2 (2)	C15—C14—C21—C16	−1.6 (3)
N1—C10—C11—C12	2.8 (4)	C10—C14—C21—C16	−179.4 (2)
C14—C10—C11—C12	179.2 (2)	C8—C9—N1—C10	−0.4 (4)
N1—C10—C11—C13	−172.8 (2)	C11—C10—N1—C9	−2.3 (4)
C14—C10—C11—C13	3.5 (4)	C14—C10—N1—C9	−179.0 (2)
C9—C8—C12—C11	−2.0 (4)	C14—C15—N3—C16	−1.7 (3)
C7—C8—C12—C11	−175.1 (2)	C22—C15—N3—C16	172.1 (3)
C10—C11—C12—C8	−0.5 (4)	C17—C16—N3—C15	−176.9 (3)
C13—C11—C12—C8	175.2 (2)	C21—C16—N3—C15	0.6 (3)
C12—C11—C13—N2	47 (22)		

### Hydrogen-bond geometry (Å, º)

Cg3 and Cg4 are the centroids of the C1–C6 and C16–C21 rings, respectively.

**Table:**

*D*—H···*A*	*D*—H	H···*A*	*D*···*A*	*D*—H···*A*
O1—H1···O2	0.82	1.91	2.596 (3)	140
N3—H3*A*···N2^i^	0.86	2.27	3.110 (4)	164
C2—H2···*Cg*3^ii^	0.90	2.93	3.656 (4)	136
C12—H12···*Cg*4^iii^	0.93	2.99	3.361 (4)	106

Symmetry codes: (i) −*x*, −*y*+2, −*z*; (ii) −*x*+3/2, *y*+1/2, −*z*+1/2; (iii) −*x*+1/2, *y*+1/2, −*z*+1/2.
