# Supplementary figures and images for: Crystal structure of 5-(5-chloro-2-hydroxy­benzo­yl)-2-(2-methyl-1H-indol-3-yl)nicotino­nitrile
Source: Acta Crystallogr E Crystallogr Commun. 2015 Oct 7;71(Pt 11):o822–3. doi: 10.1107/S2056989015018058 (PMC4645016; doi:10.1107/S2056989015018058)

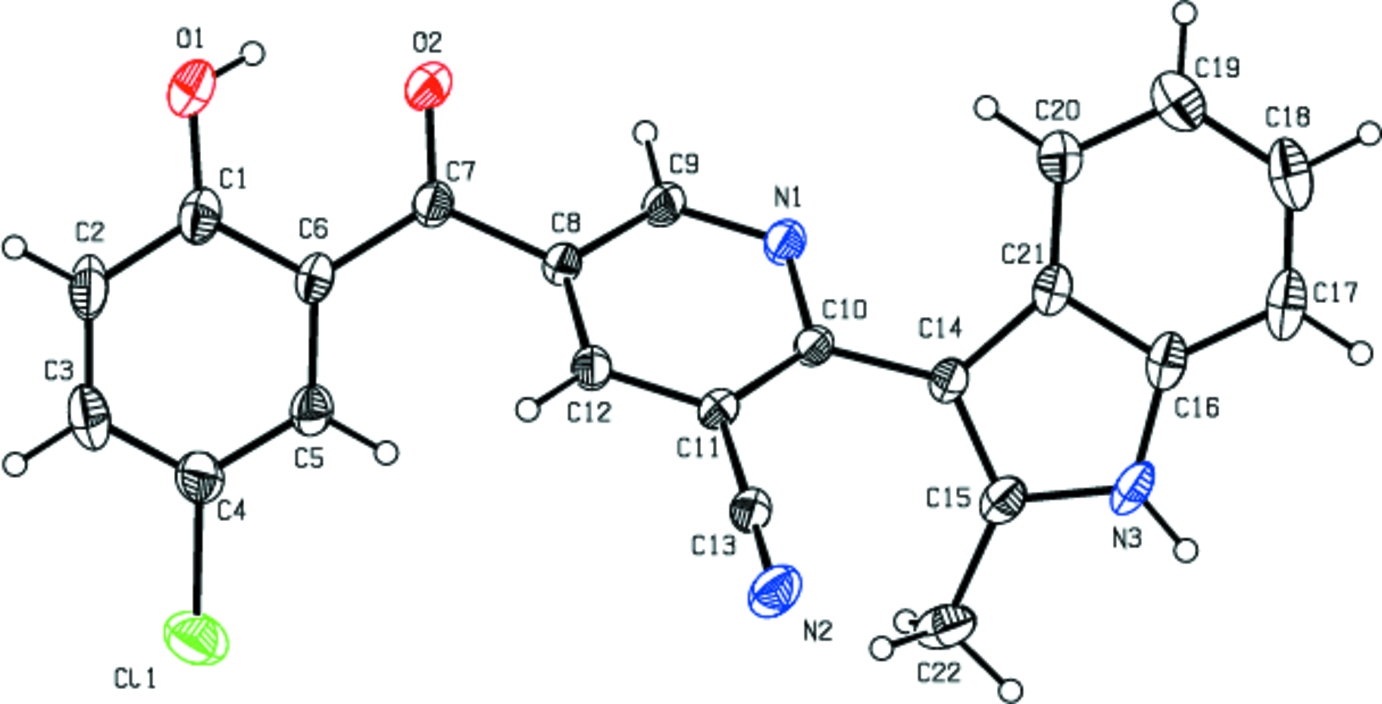

Supplement: Supplementary file 4 [file e-71-0o822-fig1.tif]

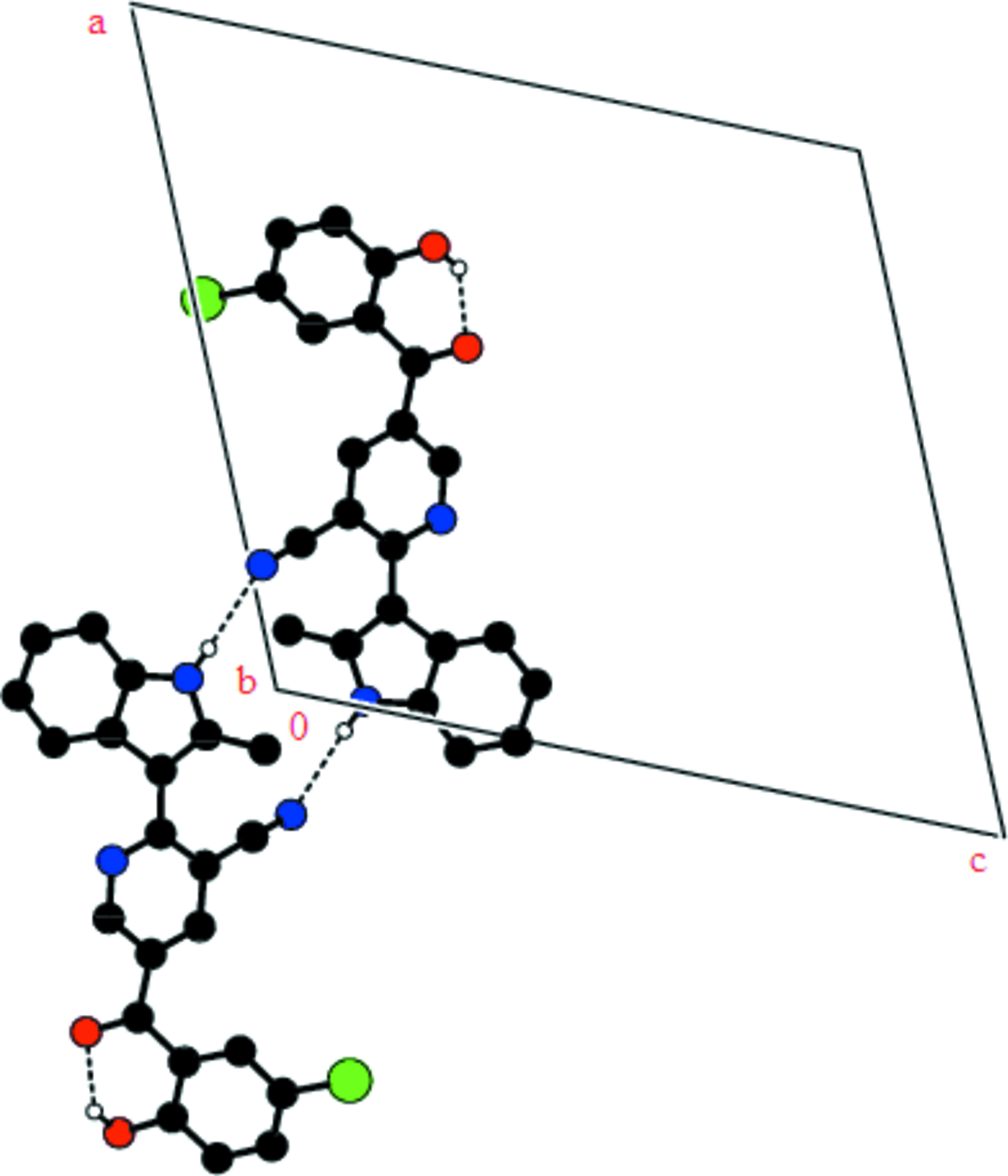

Supplement: Supplementary file 5 [file e-71-0o822-fig2.tif]
